# ABC transporters in CSCs membranes as a novel target for treating tumor relapse

**DOI:** 10.3389/fphar.2014.00163

**Published:** 2014-07-10

**Authors:** Laura Zinzi, Marialessandra Contino, Mariangela Cantore, Elena Capparelli, Marcello Leopoldo, Nicola A. Colabufo

**Affiliations:** ^1^Dipartimento di Farmacia-Scienze del Farmaco, Università degli Studi di Bari “A. Moro,”Bari, Italy; ^2^Dipartimento di Farmacia-Scienze del Farmaco, Biofordrug srl, Spin-off of University of BariBari, Italy

**Keywords:** CSCs, MDR, TICs markers, HTS, P-gp

## Abstract

CSCs are responsible for the high rate of recurrence and chemoresistance of different types of cancer. The current antineoplastic agents able to inhibit bulk replicating cancer cells and radiation treatment are not efficacious toward CSCs since this subpopulation has several intrinsic mechanisms of resistance. Among these mechanisms, the expression of ATP-Binding Cassette (ABC) transporters family and the activation of different signaling pathways (such as Wnt/β-catenin signaling, Hedgehog, Notch, Akt/PKB) are reported. Therefore, considering ABC transporters expression on CSCs membranes, compounds able to modulate MDR could induce cytotoxicity in these cells disclosing an exciting and alternative strategy for targeting CSCs in tumor therapy. The next challenge in the cure of cancer relapse may be a *multimodal strategy*, an approach where specific CSCs targeting drugs exert simultaneously the ability to circumvent tumor drug resistance (ABC transporters modulation) and cytotoxic activity toward CSCs and the corresponding differentiated tumor cells. The efficacy of suggested multimodal strategy could be probed by using several scaffolds active toward MDR pumps on CSCs isolated by tumor specimens.

## Introduction

The emerging concept about tumorigenesis is that cancer lesions are organized in a hierarchy of heterogeneous cell populations displaying different biological properties and tumorigenic potentials (Lorico and Rappa, 2011). Among these populations, only a small portion, the cancer stem cell or tumor-initiating cell (CSC/TIC) subpopulation is able to induce tumor formation and growth, leading to differentiated cells, the bulk of the tumor. In 1977 Hamburger and Salmon suggested the “*CSC theory*” by hypothesizing that cancer originates from uncommon cells, CSCs, showing pluripotency and self-renewal (Hamburger and Salmon, 1977; Boman and Wicha, 2008). In 1997 Lapidot and coworkers identified CSCs in leukemia on the basis of cell-surface-markers expression (Lapidot et al., 1994). The discovery of CSCs in leukemia and in several solid tumors, such as breast carcinoma (Al-Hajj et al., 2003; Hemmati et al., 2003; Singh et al., 2003; Carla et al., 2005; Fang et al., 2005; Xin et al., 2005; Lawson et al., 2007; Li et al., 2007; Ricci-Vitiani et al., 2007), was the proof of the *CSC theory* in cancer development.

CSC/TICs are characterized by the following properties: (a) production of all types of cells in a tumor, including CSC/TICs and non-CSC/TICs; (b) unlimited self-renewal and division capacity; (c) quiescence or slow proliferation, and (d) resistance to conventional antineoplastic therapy (Clevers, 2011). Moreover, recent studies have demonstrated that CSC/TIC phenotypes such as self-renewal and pluripotency are acquired by the activation of oncogenic genes or the inactivation of tumor suppressor genes (Baccelli and Trumpp, 2012). Since CSCs are characterized by specific surface markers, this subpopulation of cells can be isolated from mixed tumorigenic and non-tumorigenic tumor cells, by different methods of immune selection. CD44 targeting is used in the treatment of acute myeloid leukemia (AML) (Jin et al., 2006), CD24 targeting is for the treatment of colon and pancreatic cancer (Sagiv et al., 2008) and CD133 is targeted for the treatment of hepatocellular and gastric cancer (Smith et al., 2008). The current antineoplastic agents, able to inhibit bulk replicating cancer cells, and radiation treatment are not efficacious toward CSCs and, therefore, targeting these cells could be an helpful strategy for eradicating tumors more efficiently.

However, CSCs possess several intrinsic mechanisms of resistance to current chemotherapeutic drugs. Among these mechanisms, the expression of ATP-Binding Cassette (ABC) transporters family (An and Ongkeko, 2009; Calcagno et al., 2010; Fuchs et al., 2010; Clevers, 2011; Moitra et al., 2011; Pietras, 2011) and the activation of different signaling pathways such as Wnt/β-catenin signaling (Teng et al., 2010; Yeung et al., 2010; Takebe et al., 2011; Janikowa and Skarda, 2012), Hedgehog (Hh), Notch (Kobune et al., 2009; Wang et al., 2009; Zhao et al., 2009; Takebe et al., 2011; Janikowa and Skarda, 2012; Jiang et al., 2012), Akt/PKB, ATR/CHK1 survival pathways (Ma et al., 2008; Korkaya et al., 2009; Jiang et al., 2012) and constitutive activation of NF-κB are reported (Zhou et al., 2008; Liu et al., 2010).

The first of these mechanisms is the overexpression of ABC transporters such as P-glycoprotein (P-gp), Breast Cancer Resistance Protein (BCRP), and Multidrug Resistance-associated Proteins (MRPs) that use the energy of ATP hydrolysis to extrude compounds out of cells (Colabufo et al., 2010). These proteins are overexpressed in several tumors and, since responsible for drug efflux, are the main cause of MultiDrug Resistance (MDR) (Colabufo et al., 2010). Among these transporters, P-gp is the mostly studied and is localized in the luminal membrane of endothelial cells constituting the Blood-Brain Barrier (BBB), Blood-CerebroSpinal Fluid Barrier (B-CSF), and Blood-Testis Barrier (BTB); thus P-gp exerts a protective function in our body (Pharm et al., 2008; Colabufo et al., 2010). BCRP as P-gp monomer, is considered a “half-transporter” and it effluxes the same P-gp substrates (Pharm et al., 2008; Colabufo et al., 2010). MRPs differ from P-gp for the presence of an additional and specific five transmembrane domain and it efflux organic ions with high molecular weight (Pharm et al., 2008; Colabufo et al., 2010).

Since several antineoplastic drugs are substrates of ABC transporters, one of the strategy to reverse MDR is the use of inhibitors toward these pumps and their co-administration with antineoplastic agents (Perez-Tomas, 2006; Teodori et al., 2006; Gimenez-Bonafe et al., 2008). However, when antineoplastic drugs and P-gp inhibitors are co-administrated, an increased toxicity has been observed because, at the same time, the protective role of P-gp is abolished (Coley, 2010).

Over the last decade, our research group has developed P-gp ligands with different scaffolds displaying different P-gp intrinsic activities (Colabufo et al., 2008a,b, 2009, 2013; Contino et al., 2012, 2013a,b; Nesi et al., 2014). Therefore, considering ABC transporters expression on CSCs membranes, compounds able to modulate MDR activity could induce cytotoxicity in these cells disclosing an exciting and alternative strategy for targeting CSCs in tumors therapy.

## CSCs biomarkers

CSCs display several cell surface markers and their detection is useful for the identification of CSCs in tumors. In addition, the development of antibodies or antibody constructs toward these markers is also suggested as a therapeutic strategy to target CSCs (Kwon and Shin, 2013).

### CD133 and ALDH1

CD133 and ALDH1 have been identified as markers of TICs in primary human ovarian tumors (Landen et al., 2010). CD133 has been also identified as surface marker to characterize adult stem/progenitor cells in human thyroid glands (Thomas et al., 2006). ALDH1 and CD133 are also markers for the identification of CSCs in colorectal carcinoma (CRC) (Zhou et al., 2014). CD133, together with CD44, is a specific cell-surface marker of prostate cancer (Wang et al., 2013a).

### CD44, CD117, CD24

The hyaluronic acid (HA) receptor CD44 and the stem cell factor receptor CD117 are specific surface markers of ovarian CSC/TICs (Zhang et al., 2008). CD44+CD117+ cells isolated from human ovarian adenocarcinomas represent a subpopulation with an ovarian tumor-initiating capacity, that, injected in mice, induce original tumors from which they are derived (Zhang et al., 2008). CD24+ cells, isolated from patient tumor specimens, are enriched in ovarian CSC/TICs and have stem-like properties, such as chemoresistance, self-renewal and differentiation (Gao et al., 2010).

## Methods for the isolation of CSCs

Fluorescence-Activated Cell Sorting (FACS) is a magnetic cell separation method used to isolate CSCs based on the expression of specific cell surface markers, such as CD24, CD44, and CD133 (Wright et al., 2008; Takaishi et al., 2009). The detection can be performed with specific antibodies in flow cytometry, competitive ELISA (cELISA) or magnetic beads (Dobbin and Landen, 2013). Xia and coworkers have developed humanized antibodies toward the surface marker CD133 that was detected by cELISA (Xia et al., 2013). However the limitation of surface markers recognition is that the results can be ascribed to the specific studied population. Therefore, a second method, represented by the detection of the activity of a specific protein such as the membrane pump ABCG2 or ALDH1A1 enzyme, seems to be more useful than markers recognition alone. The first technique uses the DNA-binding dye Hoechst 33342, originally developed for bone marrow cells (Goodell et al., 1996). This fluorescent dye, used to identify a Hoechst-negative CSC “side population” (Kondo et al., 2004), has been successfully employed to isolate stem cells from solid tissues such as skeletal muscle, lung, liver, testis, kidney, skin, mammary gland and brain (Challen and Little, 2006). This method allows to select the cells displaying an increase in the Hoechst 33342 efflux, ABCG2-mediated, from the nucleus. The limitation of this method is due to the dye toxicity (Siemann and Keng, 1986; Erba et al., 1988).

The high ALDH activity described for CSCs suggested also this enzyme as a probe to isolate these cells. Aldefluor, an ALDH1 substrate, initially used for the isolation of hematopoietic stem cells by FACS (Storms et al., 1999; Cheung et al., 2007) and for CSCs separation in tumor tissue and cancer cell lines (Moreb, 2008; Jiang et al., 2009), has been successfully used for ALDH1 activity detection of CSCs in lung (Jiang et al., 2009), prostate (Li et al., 2010), breast (Charafe-Jauffret et al., 2010), colon (Huang et al., 2009a), and bladder (Su et al., 2010). Aldefluor contains the ALDH1 substrate BODIPY-aminoacetaldehyde (BAAA), that is converted into the fluorescent metabolite BODIPY-aminoacetate (BAA) by ALDH1 (Storms et al., 1999). BAA is then retained in living cells, because of its charge and also because Aldefluor inhibits the multidrug-resistance transporters. Therefore, Aldefluor-treated cells, with high ALDH1 activity, display high fluorescence and can be isolated by FACS into two subpopulations: ALDH-hi and ALDH-low (Moreb, 2008). This method has been validated for some cancers but it has not been widely employed because of some limitations due to cells handling that can induce stress, disrupt gene expression, and lead to altered cell physiology (Orfao and Ruiz-Arguelles, 1996). To overcome these limitations, the use of Adelfluor staining has been assessed in adherent cell cultures, that resulted in the attached-cell Aldefluor method (ACAM). ACAM displays several advantages since single-cell imaging of physiological processes in CSCs is easier in monolayers than in cells suspension or tumorsphere cultures that are usually used for growing CSCs (Fael Al-Mayhani et al., 2009; Pollard et al., 2009; Hirschhaeuser et al., 2010).

CSCs have also been usually sorted into SP cells using UV excitation (λ = 350 nm) but the limitation of this method was the cell damage caused by UV radiation exposure. Tomiyasu et al. reported a new method in which CSCs have been sorted by a violet laser (λ = 407 nm) to avoid DNA damage (Tomiyasu et al., 2014).

Another method to select CSCs is the use of molecular probes, termed aptamers, that are produced by the iterative *in vitro* selection process SELEX (Systematic Evolution of Ligands by EXponential enrichment) (Ellington and Szostak, 1990; Tuerk and Gold, 1990). Aptamers are short single-stranded oligonucleotides of DNA or RNA sequences that fold into unique secondary and tertiary structures that selectively bind the target proteins and other small molecules with high affinity and selectivity. They can be synthesized *in vitro* and therefore do not require animal or bacterial hosts for production. In recent years, in order to improve aptamers qualities and reducing time and cost of production, a number of new SELEX variants have been performed and among them, cell-SELEX uses whole living cells as target to select DNA aptamers (Shangguan et al., 2006; Sefah et al., 2010). Aptamers have many appealing features: low molecular weight, easy chemical modifications, low toxicity and immunogenicity, long shelf-life, high affinity (*K*_*d*_ value is in the picomolar to nanomolar range) and high specificity. They can be used in diagnosis, for purification processes, in drug discovery and in therapy (Proske et al., 2005). In particular, the therapeutic perspectives involve the targeting of chemotherapeutic agents, nanoparticles, drug-encapsulated liposomes and radioactive materials on cell specific biomarkers. Aptamers bind their targets with high affinity and specificity discriminating very closely related targets. The anti-theophylline aptamer is able to distinguish theophylline from caffeine, and the anti L-arginine RNA aptamer binds more strongly L-arginine than D-arginine (Jenison et al., 1994; Geiger et al., 1996). Aptamers are also useful in drug delivery strategy: 2′-fluoro- RNA aptamers have effect toward the highly expressed prostate specific membrane antigene (PSMA) (Lupold et al., 2002) and Docetaxel-encapsulated nanoparticles functionalized with a specific aptamer (A10) target PSMA expressing cells (Farokhzad et al., 2006). Another interesting example is AS1411 aptamer that recognizes Nucleolin (NCL), a multifactorial protein involved in many cellular pathways of cancer. AS1411 has been functionalized with cisplatin-liposome and evaluated in MCF-7 (NCL positive) and LNCaP (NCL negative) cell lines. The cytotoxic effects were higher in MCF-7 cells as compared to LNCaP cells (Cao et al., 2009). Moreover, specific aptamers are used in several assays to isolate, enrich and identify CSCs and for the characterization of specific markers for CSCs such as aptamers toward HA domain of CD4, RNA aptamers targeting CD133, a cell surface glycoprotein considered a universal marker of normal hematopoietic and organ-specific stem cells and aptamers identifying brain CSCs (Griguer et al., 2008; Kim et al., 2013).

## Strategies to eradicate CSCs

**Figure 1:**
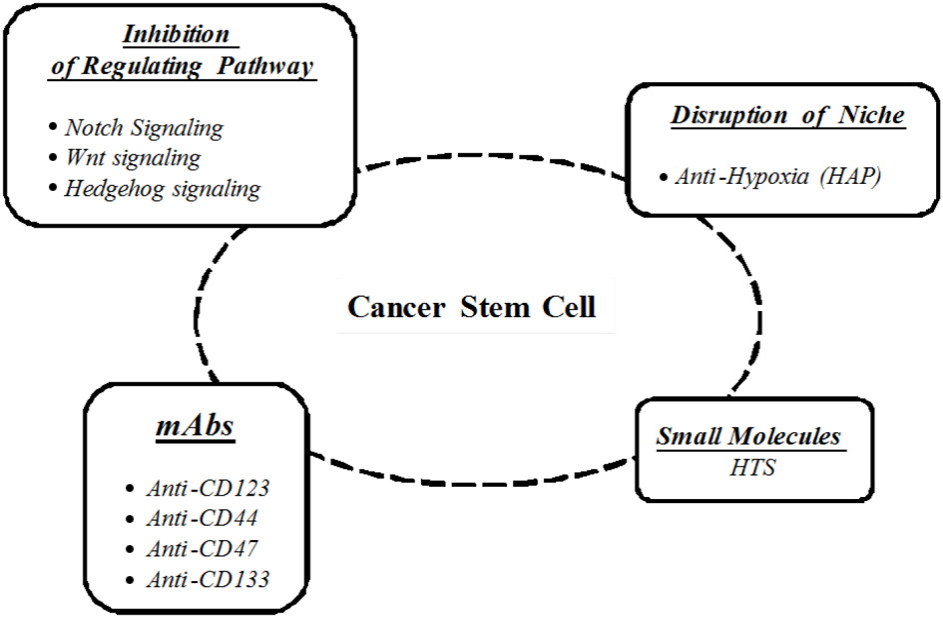
**Cancer stem cells targeting strategies**.

CSCs are responsible for tumor re-growth and potentially resistant to antitumor therapies. Therefore, the development of strategies that can act effectively against this subpopulation of cells has been envisaged, such as (Figure 1):

– Immunological therapies;
– Evaluation of genes and pathways pivotal in CSCs regulation;
– High-throughput screening (HTS) of potential inhibitory compounds;
– Regulation of tumor microenvironment.

### Immunological therapies

Since current cancer therapies fail to eradicate CSCs, causing cancer recurrence and progression, the use of monoclonal antibodies (mAbs) and antibody constructs selective for CSCs is a novel cancer therapeutic strategy. Several studies have been performed on cell surface markers that are associated with CSCs. These markers have been suggested as targets for antibodies, for antibody-drug conjugates (ADCs) in immunological therapies to overcome the side effects and the limitations of current cancer chemotherapies (Naujokat, 2014). Several mAbs, directed toward specific surface markers (Anti-CD123, Anti-CD44, Anti-CD47, Anti-CD133) or toward protein of CSCs (Anti-Notch1 and Anti-Frizzled) have been reported (Naujokat, 2014). Antibody constructs can be classified as bispecific antibodies (BsAb), targeting two different surface markers (such as BsAb-CD123 and -CD3) or as trispecific antibodies when they target three different markers (such as CD123, CD33, CD16) (Naujokat, 2014). The use of mAbs or mAbs constructs had high efficacy in tumor xenografts mice and in some clinical trials and since these markers or proteins are expressed in CSCs, they can be specifically targeted by mAbs or constructs without collateral tissue damage. Moreover, it is also possible to conjugate antineoplastic drugs to mAbs leading to ADCs. As example, Lou et al. have developed a dual-targeting drug delivery system displaying an outer layer of polymers and traditional anti-cancer drugs such as doxorubicin dispersed in the polymer (Lou et al., 2012). The internal part is represented by the ADCs since includes mAbs directed to the specific marker of CSCs (such as anti-CD133 mAb or anti-CD44 mAb), highly cytotoxic drugs (such as calicheamicin) and linkers. The inner ADCs can be released from the delivery system in tumor cells where CSCs are recognized by the specific mAb of the system and ADCs are endocytosed. Some ADCs have displayed good antitumor effects and have entered preclinical trials (Iyer and Kadambi, 2011).

PhotoChemical Internalization (PCI) is a another innovative drug delivery technology based on light-controlled cytosolic release of drugs entrapped in endosomes or lysosomes (Stratford et al., 2013).

The combination of CD133-targeting therapeutics with PCI technology, where light-activation is constrained to the tumor, has been performed to develop an immunotoxin consisting of a biotinylated anti-CD133 mAb bound to streptavidin-saporin system (Stratford et al., 2013). This combined strategy has been used in a sarcoma model system (harboring CSCs within the CD133 high population) and the results have demonstrated that PCI-based drug delivery by the CD133-receptor inhibited cells viability and growth, and the ability to form tumors *in vivo*. Since CD133 overexpression is also found in different tumors, the study performed on sarcoma model can be transferable to treatment of all solid tumors expressing CD133 (Smith et al., 2008; Waldron et al., 2011; Bostad et al., 2013).

### Regulating pathways

The inhibition of biological pathways crucial in the regulation of the renew and the maintenance of CSCs, in combination with traditional chemotherapies, is a promising strategy for the treatment of CSCs and to circumvent MDR.

#### Notch pathway

The Notch pathway is important in gene regulation and in cell differentiation processes. This pathway is involved in the pathogenesis of several human tumors such as ovarian cancer (Park et al., 2006). There are four members of the mammalian Notch receptor family (Notch 1–4) activated through a series of cleavage events. The mature Notch receptors are composed of two subunits generated from an initial cleavage event by furin-like convertase. Blaumueller et al. (1997) Notch signaling pathway activation occurs when a Notch receptor binds to ligands. This binding causes a receptor conformational change allowing a second cleavage by tumor necrosis-factor-alpha converting enzyme (TACE) (Brou et al., 2000). The following cleavage is carried out by preselenin (a γ-secretase) releasing Notch intracellular domain and activating target genes expression. Notch plays a significant role in ovarian CSCs regulation and in platinum resistance (Park et al., 2006; Takebe et al., 2011). Also, Notch 3 inhibitors increase the sensitivity of ovarian cancer to cisplatin, reducing the ovarian CSC population (McAuliffe et al., 2012).

#### Wnt pathway

The Wnt signaling pathway, regulating several processes fundamental in embryogenesis such as proliferation, differentiation, polarity, adhesion and motility, plays key roles in tumorigenesis. Indeed, the progression of several cancers is associated with specific mutations in Wnt pathway components resulting in dysregulated β-catenin-mediated gene transcription. There are two distinct pathways for transduction of Wnt signals: the “canonical” Wnt/β-catenin pathway and the “non-canonical” β-catenin-independent pathway (Clevers and Nusse, 2012). The “canonical” pathway is activated by several Wnt ligands through the binding to Frizzled (FZD) receptors and to low-density lipoprotein receptor-related proteins-5/6 (LRP5/6) co-receptors. As a result, β-catenin accumulates in the cytoplasm, translocates to the nucleus and regulates transcription of Wnt/β-catenin target genes by binding to the T-cell factor/lymphoid enhancer factor (TCF/LEF) (MacDonald et al., 2009). In the absence of Wnt signaling, β-catenin levels are regulated by a cytoplasmic destruction complex (DC). DC is a multiproteins complex composed of protein Axin, the tumor suppressor adenomatous polyposis coli protein (APC), casein kinase α (CK1α), glycogen synthase kinase 3 β (GSK3) and additional associated proteins such as the members of the PARP-family of poly-ADP-ribosylation enzymes (Tankyrases, TNKSs). Axin, the concentration-limiting component of DC, is pivotal for the stability of the β-catenin-DC. By destabilizing Axin, TNKSs directly control the levels of this protein. Therefore, TNKSs inhibition, promoting Axin stabilization, leads to β-catenin phosphorylation and degradation, attenuating Wnt signaling (Liu et al., 2002; Lee et al., 2003). For this reason, in recent years, considerable efforts have been made to identify drugs that inhibit Wnt/β-catenin signaling. Another factor exerting a fundamental role in the biogenesis of Wnt pathway is represented by a member of the Membrane-Bound O-Acyl Transferase (MBOAT) family, Porcupine (PORCN) (Tanaka et al., 2000). PORCN is an integral membrane enzyme resident in the endoplasmic reticulum, required for the lipid modification of Wnt proteins. Acylation by PORCN is essential for the proper secretion and activity of Wnt signaling and, therefore, its inhibition is an attractive therapeutic strategy in diseases with increased Wnt signaling (Chen et al., 2009).

#### Hedgehog pathway

The Hh pathway is involved in several developmental processes of cells: determination of cell fate, patterning, proliferation, survival, and differentiation (Varjosalo and Taipale, 2008). In mammals, three Hh proteins (Sonic Hh, Indian Hh, and Desert Hh) are derived from proteolysis of inactive precursor that contains its own autoprocessing domain. Hh acts by binding to 12 transmembrane glycoprotein components Patched (Ptch). In the absence of ligand, Ptch constitutively represses the activity of Smoothened (Smo), a 7-pass transmembrane spanning protein with homology to G-protein coupled receptors. When Hh ligand binds to Ptch, the repression of Smo is released and the expression and/or post-translational processing of the three GLI zinc-finger transcription factors, inducing the expression of several target genes, is modulated (Sasaki et al., 1999). Overactivation of Hh pathway is associated with cancer and emerging data from many human tumors, including glioblastoma, breast cancer, pancreatic cancer, and chronic myeloid leukemia (CML), have suggested that Hh signaling regulates CSCs (Liu et al., 2006; Bar et al., 2007; Feldmann et al., 2007; Zhao et al., 2009). The Hh signaling pathway interacts also with other pathways commonly activated in human cancers, such as the phosphatidylinositol-3-kinase (PI3K)/Akt pathway (Riobo et al., 2006).

#### PI3K/AKT pathway

PI3K/AKT is important for pluripotency maintenance of stem cells. PI3K enzymes are normally regulated by growth factors and are useful to phosphorylate phospholipids on the plasma membrane (Hennessy et al., 2005). In addition, it has been suggested that PI3K/Akt activation pathway is required for CSCs maintenance and viability in breast cancer, prostate cancer, and brain tumor (Zhou et al., 2007; Hambardzumyan et al., 2008; Dubrovska et al., 2009). The PI3K/Akt pathway can also modulate functions of ABC transporters through various mechanisms. Inhibition of the PI3K/Akt pathway causes BCRP internalization in hematopoietic stem cells and glioma stem-like cells. Hence, the PI3K/Akt pathway can be an attractive target for cancer therapy (Mogi et al., 2003; Bleau et al., 2009).

### High-throughput screening (HTS)

The development of agents active toward CSCs population may be useful in the treatment of patients with recurrent cancer. HTS allows to identify potential compounds targeting specifically CSCs. In recent years, several studies have been carried out and in one of these studies the chemical library Microsource Spectrum collection of 2000 compounds has been screened to identify therapeutic agents inhibiting Glioblastome Multiforme stem cells (GSCs). This library has been selected based on the structural and biological diversity and consisted of FDA approved drugs, compounds in the late-phase clinical trials, experimental drugs and natural products. Among these, Disulfiram, a clinically approved drug for the treatment of alcoholism, was identified as a potent inhibitor of multiple patient-derived GSCs (Germain et al., 2013).

Another study has been carried out in order to identify agents able to selectively inhibit a cell-line model of breast CSCs screening over 300,000 compounds of the Molecular Libraries Small Molecules Repository (MLSMR) from the National Institute of Health (NIH). Among these compounds, a cinnamide analog displayed 20-fold selective inhibition of the breast CSC-line cell (HMLE_sh_Ecad) over the control (HMLE_sh_eGFP) (Hothi et al., 2012).

In another study, HTS of 825 potential drugs (the National Cancer Institute's “Mechanistic Set” library) toward ovarian CSCs and the subsequent identification of compounds displaying potential activity as ovarian CSC therapeutic agents is reported. Several compounds were classified as inhibitors and, among these, 5 compounds FDA-approved (dactinomycin, plicamycin, vinblastine, vincristine, and mepacrine) were identified as anticancer drugs (Mezencev et al., 2012).

### Tumor microenvironment

**Chart 1:**
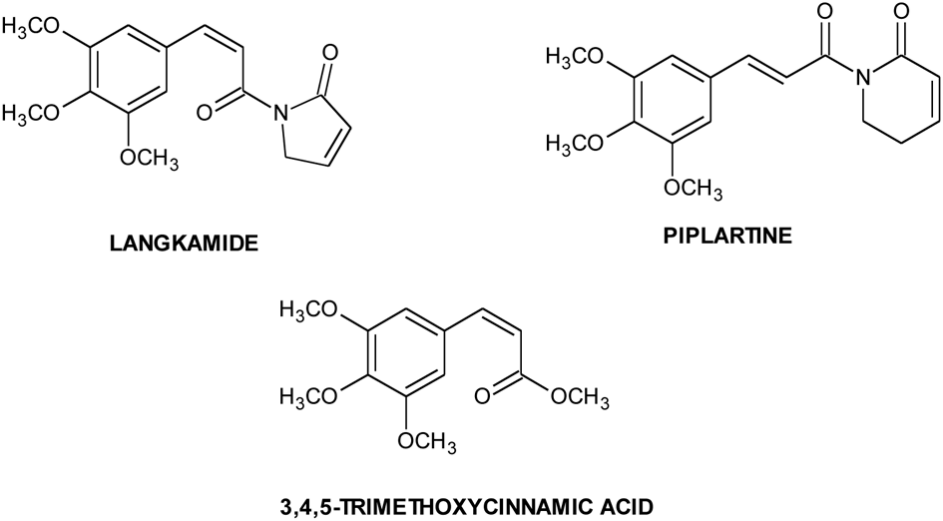
**Natural HIF inhibitors**.

It has been proposed that CSCs are maintained by their surrounding microenvironment called “niche.” Niche, important for CSCs sustaining, is composed by immune and stromal cells, blood vessels and extracellular matrix components. Biological processes, such as inflammation, hypoxia and angiogenesis, are important for CSCs, and signaling from this microenvironment is crucial for the activation of pathways involved in the maintenance of TICs functions (Cabarcas et al., 2011; Hanahan and Weinberg, 2011). Tumor hypoxia is implicated in the development of resistance to many conventional chemotherapeutic agents. It induces a decreased sensitivity to apoptotic and other cell-death signals, and increased signaling promoting angiogenesis, proliferation and systemic metastasis capacity (Feldmann, 2001; Kunz and Ibrahim, 2003). The initial response of cancer cells to hypoxia is the activation of hypoxia responsive transcription factors (HIFs) that regulate a number of genes involved in glucose metabolism, cell survival, erythropoiesis, stem cell maintenance, angiogenesis and resistance to chemotherapy and radiotherapy (Majmundar et al., 2010). Molecules like langkamide, piplartine and 3,4,5-trimethoxycinnamic acid (Chart 1), displaying HIF-2 inhibitory activity, may be used for targeting HIFs mediated regulation of CSCs (Bokesch et al., 2011). Another important transcription factor, involved in tumor growth, progression, and in the resistance to chemotherapy, is NFkB: this factor and HIF1α together regulate transcription of thousand genes controlling vital cellular processes (Gupta et al., 2010).

## Molecules active toward CSCs

### Porcupine and TANKSs inhibitors

In the last years, considerable efforts have been made to identify drugs able to inhibit Wnt/β-catenin signaling, either by blocking Wnt secretion (PORCN inhibitors) or by interfering with β-catenin, by binding its transcription factor targets (TANKSs inhibitors).

**Chart 2:**
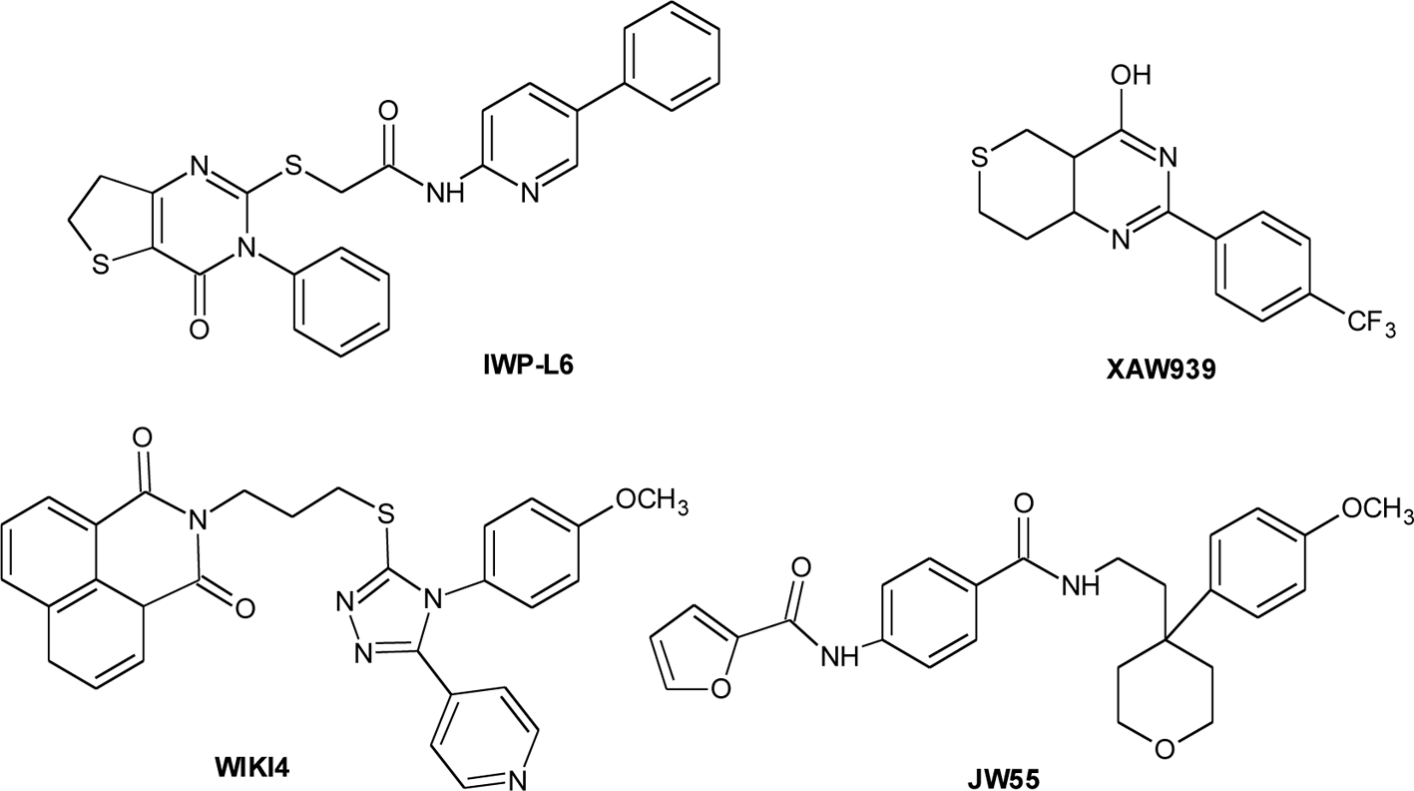
**Porcupine and Tankyrase inhibitors**.

Starting from four IWP ligands, additional PORCN inhibitors have been identified, that are characterized by two structural motifs of IWPs (phthalazinone and pyrimidinone moieties) interacting with PORCN and important for the activity. The lead optimization step led to a significant improvement in activity and to the identification of IWP-L6 (Chart 2) as a new sub-nanomolar PORCN inhibitor (Chen et al., 2009; Wang et al., 2013b). The first TANKSs inhibitor, identified by Huang et al. through a chemical genetic screen, was XAV-939 (Chart 2) (Huang et al., 2009b). It selectively affects β-catenin-mediated transcription, by stimulating its degradation by stabilizing Axin. By HTS, JW55, IWR-1, and WIKI4 (Chart 2) as specific tankyrases inhibitors have been discovered (Chen et al., 2009; James et al., 2012; Waaler et al., 2012).

### HAP: hypoxia-activated bioreductive prodrugs

The hypoxic microenvironment of solid tumors has attracted significant attention as target for the development of novel therapeutics for cancer treatment. A new class of drugs, the hypoxia activated prodrugs (HAP), has been designed for selective activation under low oxygen conditions, typical of many solid tumors. These are prodrugs that are enzymatically converted into active metabolites (effectors) by endogenous enzymes under the hypoxic conditions that prevail in tumors (Rauth et al., 1998). Taking into account the different activation profile, HAP could be classified into two groups: (1) Class I HAP (such as benzotriazine *N*-oxides tirapazamine and SN30000), activated under relatively mild hypoxia; (2) Class II HAP (such as the nitro compounds PR-104A or TH-302), activated only under extreme hypoxia (Yin et al., 2012; Foehrenbacher et al., 2013). Several hypoxia-specific prodrugs are in various stages of clinical development, although no registered agents have been used in clinical therapy. Moreover, the bioreductive compounds are classified in five classes: (1) aromatic *N*-oxides; (2) aliphatic *N*-oxides; (3) nitro(hetero)cyclic compounds; (4) quinones; (5) metal complexes.

**Chart 3:**
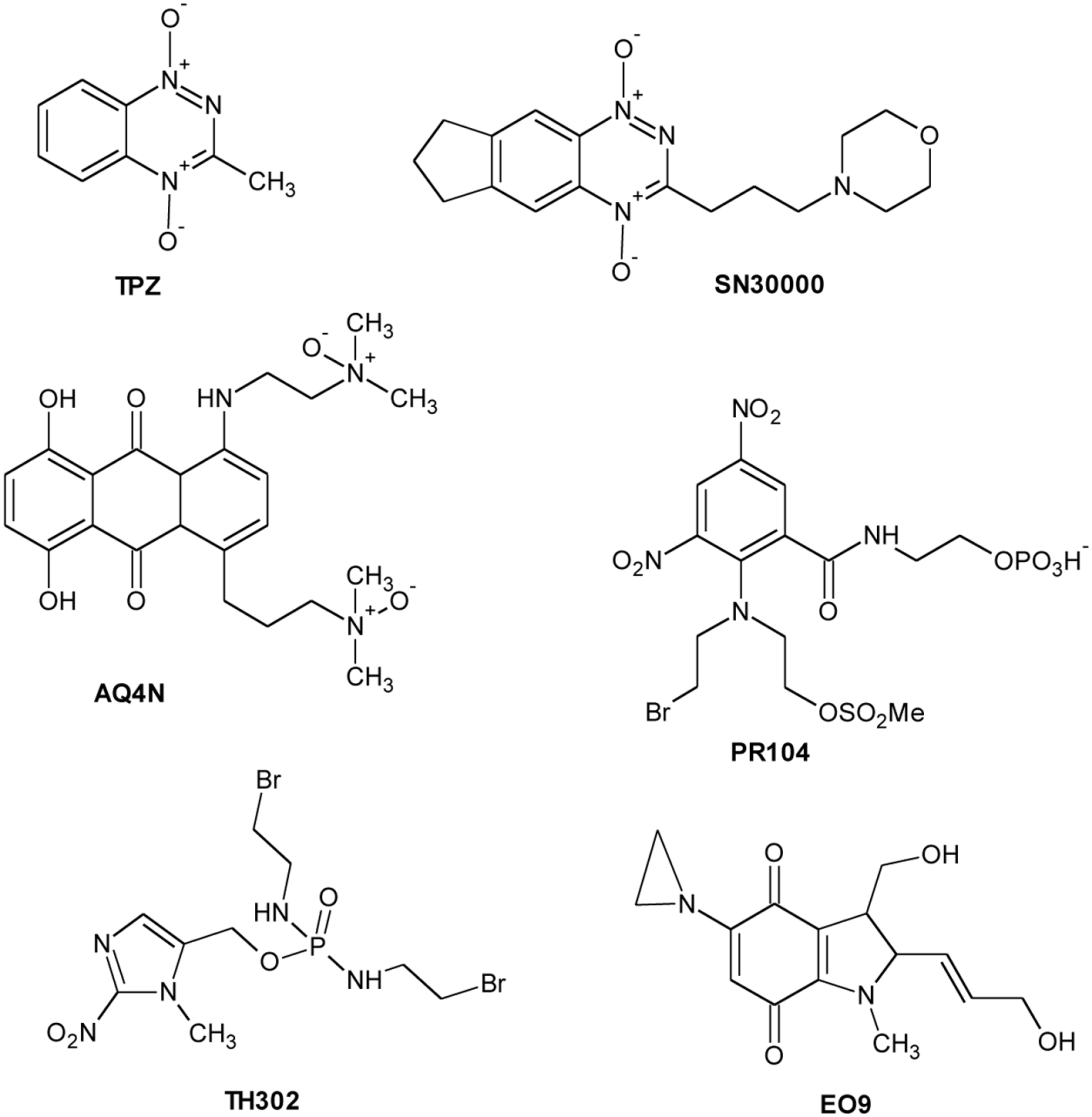
**Hypoxia Activated Prodrugs (HAP)**.

The *N*-oxide tirapazamine (TPZ, SR4233) (Chart 3) has been the most extensively evaluated compound in the clinic to date. TPZ exhibited up to 300-fold higher toxicity under anoxic conditions than aerobic conditions *in vitro* (Zeman et al., 1986). TPZ undergoes one-electron reduction to generate the corresponding radical able to produce DNA breaks and lesions (Shinde et al., 2010). Although TPZ has been evaluated in a number of phase II clinical trials (Marcu and Olver, 2006), the results have not been translated into increased efficacy over conventional treatment in phase III clinical trials (Rischin et al., 2010). SN30000 (Chart 3) is a TPZ analog with improved pharmacokinetic properties and is presently scheduled to enter phase I clinical trials (Hicks et al., 2010).

*N*-oxide banoxantrone (AQ4N) (Chart 3) is metabolized under hypoxia giving a high affinity DNA intercalating agent, AQ4, that inhibits topoisomerase II (Patterson et al., 1994). In preclinical studies, AQ4N, combined with radio- or chemo-therapy, demonstrated significant increasing activity (Patterson et al., 2000).

PR-104 (Chart 3), currently in phase II clinical trials, is a water-soluble phosphate ester pre-prodrug that rapidly is hydrolyzed *in vivo* to the prodrug PR-104A, a dinitrobenzamide mustard that is reduced to *p*-hydroxylamine and *p*-amine metabolites by various oxidoreductases (Singleton et al., 2009). Another promising compound, TH-302 (Chart 3), is a 2-nitroimidazole-based nitrogen mustard prodrug. The reduction in hypoxic cells to 2-nitroimidazole and the subsequent bromoisophosphoramide mustard affects DNA repair pathways (Meng et al., 2012). TH-302, in combination with chemotherapy, is currently in phase II and III clinical trials.

Quinone compounds (porfiromycin, RH1, and apaziquone EO9) (Chart 3) show greater selectivity toward hypoxic cells where their activation under hypoxia is carried out by one-electron reductases (Saunders et al., 2000; Phillips et al., 2013; Guise et al., 2014). Finally, although metal complexes could be potentially used as hypoxia-selective agents, none have been developed for clinical use so far.

### Miscellaneous

**Chart 4:**
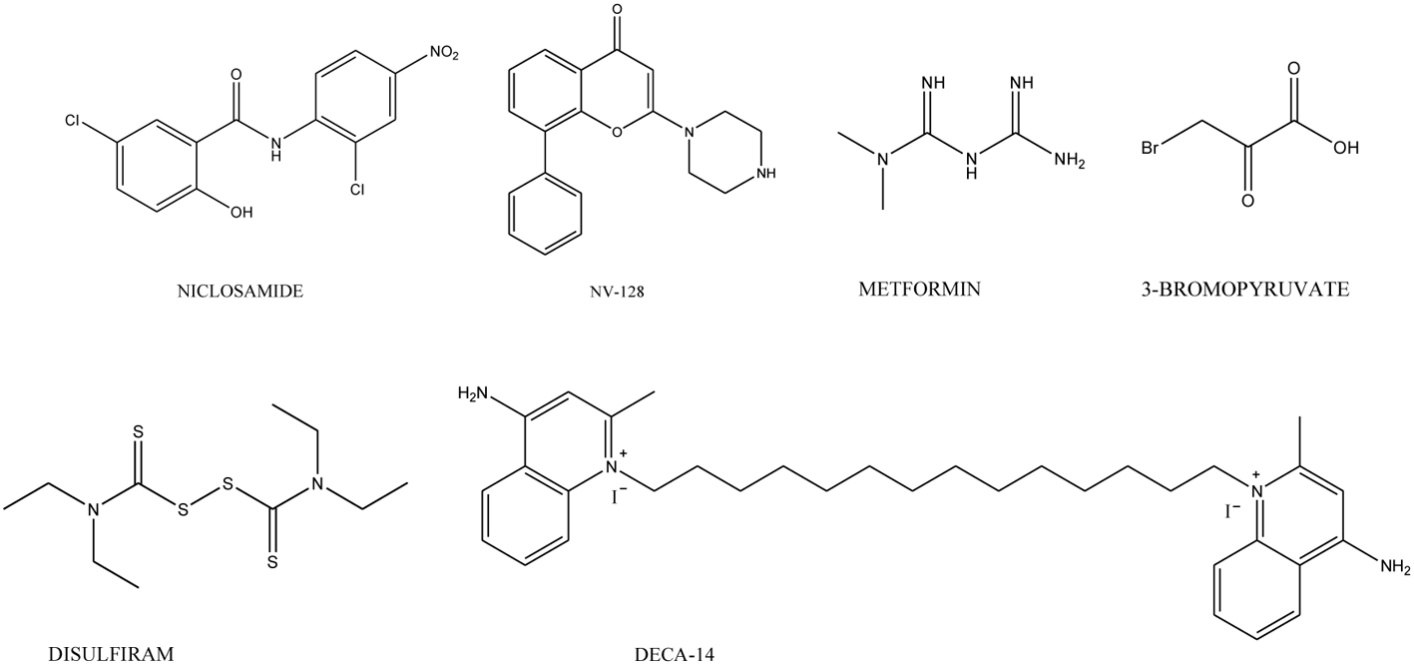
**Agents targeting CSCs**.

Several molecules, with unrelated chemical structures, are active toward CSCs. Among these, the antihelmintic niclosamide (Chart 4) selectively targets ovarian CSCs, and its effect is associated with the inhibition of metabolic pathways related to redox regulation (Yo et al., 2012). Metformin (Chart 4), an anti-diabetic drug, selectively targets CSCs in multiple types of cancer including prostate, lung and breast (Hirsch et al., 2009; Shank et al., 2012) and, in combination with chemotherapeutic agents (such as doxorubicin, paclitaxel, and carboplatinum) inhibits tumor growth (Gotlieb et al., 2008). This drug also displays an anti-proliferative and pro-apoptotic effects on ovarian CSCs *in vitro* (Yasmeen et al., 2011). Retinoids, in co-administration with cis-platinum, affect ovarian CSCs, whereas carboplatinum alone is not active (Whitworth et al., 2012). Ericalyxin B, a diterpenoid isolated from *Isodon eriocalyx* displaying antitumor effects *via* multiple pathways, and 3-Bromopiruvate target ovarian CSCs inducing cell death (Leizer et al., 2011; Wintzell et al., 2012). Dactinomycin and Plicamycin, two FDA-approved CSCs-inhibitory compounds, are used in the treatment of several cancers including gestational trophoblastic neoplasia, Wilms' tumor, testicular cancer and hypercalcemia associated with advanced malignancy (Kennedy, 1970; Perlia et al., 1970; Lewis et al., 1972; Frei, 1974; Fraschini et al., 2005; Lee et al., 2006). Disulfiram (Chart 4), used in alcoholism treatment, is able to reduce cell growth and self-renewal of glioblastoma stem cells resistant to temozolomide *in vitro* by activating apoptotic pathways that modulate Bcl-2 family (Liu et al., 2012). DECA-14 (Chart 4), an analog of dequalinium (DECA-10), an antimicrobial agent and Rapamycin were identified as neuroblastoma (NB) TIC-selective agents. Both compounds induce CSCs death *in vitro*, reduce NB xenograft tumor weight *in vivo* and decrease self-renewal in treated tumors (Smith et al., 2010). Furthermore, NV-128 (Chart 4), an isoflavone derivative, induces cell death in chemoresistant CSCs population by inhibiting mitochondrial function and activating cell death pathways (Alvero et al., 2011).

## CSC and MDR: repositioning strategy

The failure of cancer therapy is often due to tumor recurrence after chemotherapy because in several cancers a residual pool of CSCs remains (Dean et al., 2005). TICs that survive after therapy evolve in a population of chemoresistant cells able to sustain the growth of a more aggressive tumor. Among all the protective mechanisms for CSCs, ABC proteins overexpression is one of the mostly reported (An and Ongkeko, 2009; Calcagno et al., 2010; Fuchs et al., 2010; Clevers, 2011; Moitra et al., 2011; Pietras, 2011). There are two models useful to explain the connection between MDR and CSCs: (i) the original MDR model and (ii) the acquired MDR model (Holohan et al., 2013). The first model proposes that, after exposure to the chemotherapeutic agent, only CSCs expressing ABC transporters repopulate the tumor. The second suggests that, after chemotherapy, only CSCs survive and this population of survival cells, after mutations, originates new and more aggressive drug-resistant cell phenotypes (Holohan et al., 2013). The combination of CSCs targeting agents with novel or conventional cytotoxic drugs could lead to a potentiated effect. Flavonoids, a large family of polyphenolic molecules, modulate MDR transporters and inhibit CSCs growth (Colabufo et al., 2008c). Flavonoids anticancer properties are due to their antimitotic activity and to the inhibition of several enzymes. Recently, it has been suggested that flavonoids inhibit the function of ABC transporters such as P-gp, MRPs, and BCRP. Structural requirements for the interaction with these pumps are hydrophobic character and planarity. Flavonoids show low toxicity but, in the meantime, display a broad spectrum of biological activities (Colabufo et al., 2008c). Moreover, several studies have pointed out that flavonoids are able to affect CSCs. Luteolin, Casticin and 8-Bromo-7-methoxychrysin (BrMC) are active on glioma stem-like cells (Feng et al., 2012). BrMC is active on hepatocellular carcinoma (Hep-G2 cell line) where it induces apoptotic cell death by involving ROS products (Yang et al., 2010). LY294002, a PI3K specific inhibitor, blocks osteosarcoma CSCs cell cycle (G0/G1) in a dose-dependent manner inducing apoptosis by preventing phosphorylation of PKB/Akt *via* PI3K phosphorylation inhibition. Several studies show that this compound also inhibits BCRP, P-gp, and MRP1 highly expressed in stem cells. LY294002 reverses MDR in CSCs overexpressing BCRP by internalizing this pump (Imai et al., 2012). LY294002 competitively inhibits MRP1-mediated doxorubicin efflux in drug-resistant HT29RDB colon carcinoma cells. Sensitization was not restricted to doxorubicin, but it was also observed in cells treated with cisplatin, topotecan, and mitoxantron (Abdul-Ghani et al., 2006). Moreoever, LY294002 antagonizes transport activity of P-gp by reducing the degree of vincristine resistance in L1210/VCR mouse leukemic cell lines in a concentration-dependent manner (Barancík et al., 2006). However, the simultaneous inhibition of several cellular signaling pathways, transporters and channels may cause severe side effects.

Salinomycin, a polyether ionophore antibiotic isolated from *Streptomyces albus*, is able to affect CSCs in gastric cancer, lung adenocarcinoma, osteosarcoma, colorectal cancer, squamous cell carcinoma (SCC) and prostate by interfering with ABC transporters and CSC crucial pathways (Dong et al., 2011). The combination of salinomycin and the antibody for the anti-human epidermal growth factor receptor 2 (anti-HER2), trastuzumab, was found more efficacious than trastuzumab single-treatment in MCF-7-derived breast CSCs and HER2-expressing breast cancer cells. In colorectal cancer and in SCC, salinomycin, but not oxaliplatin or cisplatin, is able to reduce the portion of CSCs (Basu et al., 2011; Dong et al., 2011; Tang et al., 2011; Wang, 2011; Zhi et al., 2011; Ketola et al., 2012; Oak et al., 2012). Moreover, salinomycin is active toward human AML CSCs, because it overcomes ABC transporter-mediated MDR and apoptosis resistance and inhibits P-gp/MDR1 in different cancer cells (Fuchs et al., 2010; Riccioni et al., 2010).

## Conclusions and perspectives

Although several items about CSCs theory remain open, there is a large evidence demonstrating that CSCs are essential to initiate and maintain tumors. CSCs are involved in tumor re-growth and are resistant to conventional anti-cancer therapy because of several mechanisms. Among them, the overexpression of ABC transporters in CSCs membranes represents a novel target for eradicating tumor relapse. To date, several strategies have been employed to isolate, target CSCs and circumvent MDR: (i) the development of sensitive biomarkers and targeted-aptamers; (ii) the evaluation of CSCs microenvironment; (iii) the study of the genes and pathways involved in CSCs regulation; (iv) evaluation of known and new molecules able to affect both TIC populations and ABC transporters. Therefore, the next challenge in this field may be a *multimodal strategy*, an approach in which specific CSCs targeting drugs exert simultaneously the ability to circumvent tumor drug resistance (ABC transporters modulation) and cytotoxic activity toward CSCs and the corresponding differentiated tumor cells. The efficacy of this suggested multimodal strategy will be probed by using several scaffolds active toward MDR pumps on CSCs isolated by tumor specimens.

### Conflict of interest statement

The authors declare that the research was conducted in the absence of any commercial or financial relationships that could be construed as a potential conflict of interest.
